# Case Report: Take a Second Look: Covid-19 Vaccination-Related Cerebral Venous Thrombosis and Thrombotic Thrombocytopenia Syndrome

**DOI:** 10.3389/fneur.2021.763049

**Published:** 2021-11-22

**Authors:** Tobias Braun, Maxime Viard, Martin Juenemann, Tobias Struffert, Frank Schwarm, Hagen B. Huttner, Florian C. Roessler

**Affiliations:** ^1^Department of Neurology, University Hospital Giessen, Giessen, Germany; ^2^Department of Neuroradiology, University Hospital Giessen, Giessen, Germany; ^3^Department of Neurosurgery, University Hospital Giessen, Giessen, Germany

**Keywords:** cerebral venous thrombosis, Covid-19-vaccination, ChAdOx1 nCoV-19 vaccine, thrombotic thrombocytopenia syndrome, vaccine-induced thrombotic thrombocytopenia

## Abstract

We present two cases of ChAdOx1 nCov-19 (AstraZeneca)-associated thrombotic thrombocytopenia syndrome (TTS) and cerebral venous sinus thrombosis (CVST). At the time of emergency room presentation due to persistent headache, blood serum levels revealed reduced platelet counts. Yet, 1 or 4 days after the onset of the symptom, the first MR-angiography provided no evidence of CVST. Follow-up imaging, performed upon headache refractory to nonsteroidal pain medication verified CVST 2–10 days after initial negative MRI. Both the patients received combined treatment with intravenous immunoglobulins and parenteral anticoagulation leading to an increase of platelet concentration in both the individuals and resolution of the occluded cerebral sinus in one patient.

## Introduction

In the current SARS-Cov2-pandemic, cerebral venous sinus thrombosis (CVST) associated with thrombotic thrombocytopenia syndrome (TTS) following vaccination with vector vaccines (by AstraZeneca- and Johnson & Johnson) is an intensely studied and hotly debated topic.

Here, we report two cases of TTS-associated CVST. Both the patients had verified TTS (according to the *American Society of Hematology 2021* guideline), presented with refractory headache, and were investigated for CVST using CT and MR angiography. The initial scans of both patients were normal without evidence of CVST. Strikingly, follow-up imaging performed because of persistent headache, a few days after initial imaging, revealed significant CVST.

The report of this phenomenon is harboring significant implications for clinical routine. We believe this finding is unlikely to be unique and may potentially be overseen in current out-patients and in-hospital management of patients with TTS presenting with postvector-vaccination headache.

## Case Descriptions

**Figure 1:**
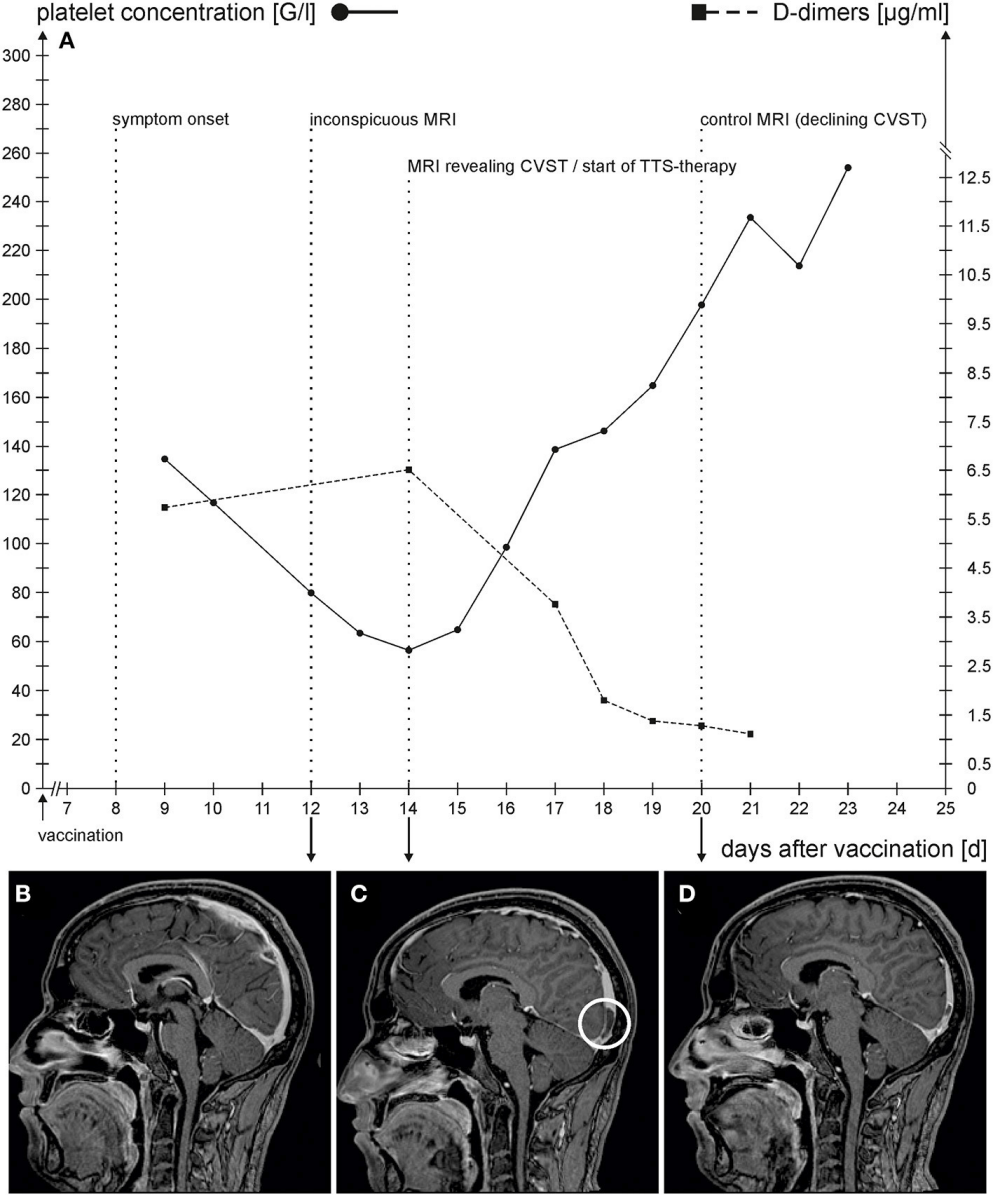
**(A)** Time course of symptom onset, laboratory findings of platelet count (solid line)/D-Dimer (dashed line), and imaging studies. The x-axis represents the number of days after vaccination. **(B–D)** show sagittal contrast-enhanced T1-weighted MRI-sequences; **(B)** no evidence of thrombosis in the sagittal superior sinus at day 12 following vaccination; **(C)** thrombosis of the sagittal superior sinus (circle) at day 14 following vaccination; **(D)** regression of thrombosis at day 20 following vaccination.

We present a 21-year-old male who had no relevant prior medical history or medication. A few hours after the first vaccination with ChAdOx1 nCov-19-vaccine, he developed flu-like symptoms with fever (38.0°C) and headache that lasted for 2 days. As the symptoms reappeared 8 days after vaccination, he was presented the following day to our hospital, with a complaint of malaise and fever. On physical examination, he showed no signs or symptoms. Laboratory testing revealed thrombocytopenia (135 G/l), elevated C-reactive protein (CRP) value (103 mg/l), and increased D-dimers (5.83 μg/ml) (Figure 1A depicts the time course of symptoms and relevant laboratory results). Covid-PCR testing was negative. Chest X-ray, urine status, and blood cultures showed no evidence of a specific infection, and the patient was started on empiric therapy with ampicillin/sulbactame. Because of elevated D-dimers and thrombocytopenia, he received weight-adjusted anticoagulation with enoxaparin. Diagnostic workup revealed no evidence of pulmonary artery embolism or thrombosis of peripheral veins. Cranial MRI with venous angiography was performed because of headache on day 12 following vaccination, providing insignificant findings (Figure 1B).

With steady decreasing platelet concentration, enoxaparin was stopped after 4 days of therapy. The patient was discharged without medication on day 13 following vaccination with the diagnosis of infection of unclear etiology and differential diagnosis of a protracted vaccination reaction. One day later, the attending physicians were notified of a positive anti-platelet factor-4-(PF4)-ELISA. The HIPA-test, on the other hand, was negative with heparin but positive with AZD1222. Therefore, heparin-induced thrombocytopenia (HIT) type 2 was excluded by laboratory testing. The patient was immediately readmitted. The diagnosis of vaccination-related TTS was confirmed by flow cytometry. The patient complained of no new symptoms apart from the persisting headache. Because of the non-resolving headache, cerebral MRI was repeated (Figure 1C). There was thrombosis of the superior sagittal sinus, starting above the confluence and extending over approximately 29 mm to the inside of the right transverse sinus. Ischemia or intracerebral hemorrhage were not present. Meanwhile, the platelet count had reached its lowest value (57 G/l). D-dimers had increased to a maximum value of 6.63 μg/ml.

Anticoagulation with fondaparinux of 7.5 mg/d was started immediately and weight-adjusted intravenous immunoglobulins were applied for 2 days. Further course was without complications with an adequate increase in the platelet count. A follow-up of native cerebral CT examination, 16 days after vaccination, provided normal findings consistent with age. Twenty days after vaccination, cranial MRI with venous angiography showed partial recanalization of thrombosis (Figure 1D). A residual thrombus was found in the sagittal sinus beginning above the confluence measuring approximately 18 mm. From day 20 after vaccination, the headache was resolved. Anticoagulation was switched to dabigatran of 150 mg, two times, and the patient was discharged.

**Figure 2:**
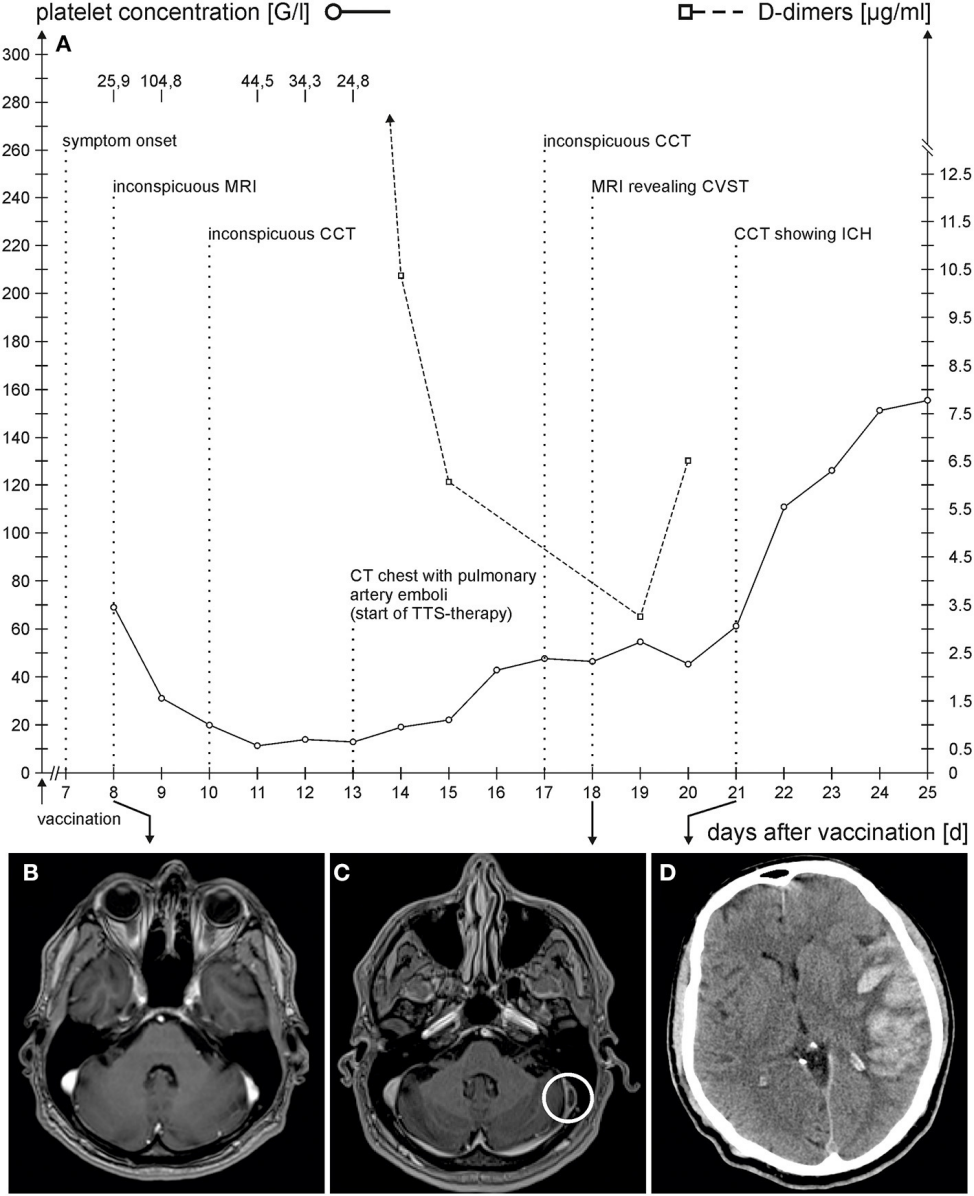
**(A)** Time course of symptom onset, laboratory findings of platelet count (solid line)/D-Dimer (dashed line), and imaging studies. The x-axis represents the number of days after vaccination. **(B,C)** show axial contrast-enhanced T1-weighted MRI-sequences: **(B)** no evidence of thrombosis in the left-sided lateral transverse sinus at day 8 following vaccination; **(C)** thrombosis in the left-sided lateral transverse (circle) sinus at day 18 following vaccination; **(D)** axial cranial CT showing a left-sided, space-occupying atypical intracranial hemorrhage (ICH) with subarachnoidal hemorrhage at day 21 following vaccination.

The second patient was a 63-year-old male who was presented under the suspicion of meningitis to a tertiary hospital. There was no relevant medical history or medication. He had experienced flu-like symptoms following the first ChAdOx1 nCov-19-vaccination 8 days ago. The day before, he experienced fever (39 °C), blurred vision, and headache. His general practitioner (GP) had diagnosed meningism and therefore started treatment with cefuroxime. Neurological examination was unremarkable. However, on the day of presentation, elevated D-dimer levels (25.9 μg/ml), thrombocytopenia (69 G/l), and CRP elevation (52.4 mg/l) were apparent (Figure 2A depicts the time course of symptoms and relevant laboratory results). Cranial MRI with venous angiography was performed and yielded insignificant findings (Figure 2B). A lumbar puncture could not be performed because of thrombocytopenia. Laboratory changes were attributed to the vaccination, and the patient was discharged.

The following day, he was presented to a primary hospital with mild retroauricular pain on coughing, blurred vision, and a persisting headache. Clinical examination revealed no findings. Because of thrombocytopenia (31 G/l) and D-dimer elevation (104.8 μg/ml), the patient was admitted. The following day, a cranial CT with venous angiography was performed, which yielded normal findings. Because of the pathologic coagulation, parameters with simultaneous lack of evidence of manifest thrombosis (inconspicuous findings in venous CT-angiography, duplex sonography of peripheral veins, and abdominal sonography), anticoagulation with 7.5 mg of fondaparinux was initiated, which was discontinued 2 days later in favor of 2.5 mg of apixaban two times. A contrast-enhanced CT of the chest was performed on day 13 after vaccination when the patient complained of dyspnea, which showed bipulmonary peripheral pulmonary artery emboli. Full anticoagulation with apixaban (2 times, 10 mg) and 2 days of intravenous administration of weight-adjusted immunoglobulins were started.

On day 14 after vaccination, an immunologic examination confirmed TTS (positive antiplatelet factor-4-(PF4)-ELISA; HIPA-test and flow cytometry were positive with AZD1222 but was negative for HIPA-test with heparin). A new cranial CT was performed on the 17th day after vaccination because of headache and vertigo and showed no relevant changes. One day later, cranial MRI with venous angiography showed left-sided thrombosis in the internal jugular vein, sigmoid sinus, and lateral portion of the transverse sinus (Figure 2C). In addition, ischemia could be detected in the left cerebellar and the right high parietal and parietooccipital cortex. The patient was transferred to our hospital. On admission, neurological examination was insignificant. Anticoagulation with apixaban was switched to partial thromboplastin time (PTT)-controlled argatroban.

After 3 days, the patient vomited and presented anisocoria, right-sided hemiplegia, and aphasia. Cranial CT revealed a left-sided, space-occupying atypical intracranial hemorrhage (ICH) suitable for stasis bleeding in sinus thrombosis with concomitant subarachnoid hemorrhage (SAH) and marked midline shift (Figure 2D). On the same day, left-sided hemicraniectomy was performed. Postoperative cranial CT showed a regredient space-occupying effect of ICH with unchanged sinus thrombosis. To treat TTS, we administered 40 mg of dexamethasone and weight-adjusted immunoglobulins for 2 days. On the following day, the clinical condition of the patient declined. Despite the resumption of argatroban, mild hypothermia (36°C), administration of hypertonic saline, and deep analgesia, subsequent CT follow-up showed an increase in hemorrhage and perifocal edema with an increase in midline shift. On day 24 after vaccination, the patient had wide pupils and subfalcial and uncal entrapment and extracranial herniation throughout the craniectomy defects were visible on CCT. The patient died the next day.

## Discussion

Cerebral venous sinus thrombosis is an uncommon disease and occurs in younger patients. Risk factors are female gender, intake of oral contraceptives or hormone replacement therapy, pregnancy and puerperium, obesity, smoking, and thrombophilia. Symptoms usually develop gradually and consist of headache, focal symptoms, encephalopathy, and epileptic seizures. When CVST is suspected, thrombosis of cerebral sinus or veins can be identified by venous angiography with contrast-enhanced CT or MRI. Intracerebral hemorrhages, as a result of venous infarction, can be detected as well. In patients with confirmed CVST, anticoagulation with unfractionated or low molecular weight heparin is started, even in the presence of intracerebral hemorrhage. After initial parenteral anticoagulation, oral anticoagulation (usually vitamin k antagonists) is initiated for 3 to 12 months (1). Recently, dabigatran was identified as a safe alternative (2). Venous recanalization is achieved in 85% of patients, but there is only limited data on the temporal profile (3).

Cerebral venous sinus thrombosis following SARS-Cov2-vaccination has been reported following SARS-Cov2-vaccines in case reports and registers. It has also been reported in conjunction with messenger RNA (mRNA) vaccination against SARS-Cov2, but only in patients with vector vaccines, TTS was detected. CVST following mRNA-vaccination has been discussed as a background coincidence (4). In a German register, the incidence of CVST was significantly higher following AstraZeneca-vaccination as compared to the pre-SARS-Cov2-vaccination period, especially in women (5). A systematic review identified headache as the most common presenting symptom. Symptoms occurred within 1 week after the first dose of vaccination. Intracerebral hemorrhage or subarachnoid hemorrhage (SAH) was reported in 49% of the patients (6).

The phenomenon of thrombocytopenia and or HIT was retrospectively assessed in a database of 865 patients with CSVT from 1987 to 2018. Thrombocytopenia was reported in 8.4% and HIT in 0.1% of the patients. No patient had anti-PF4-antibodies (7). The rarity of these findings underlines the distinct type of vaccine-associated CVST. High mortality was described when this phenomenon occurred for the first time in literature (8). The presence of intracranial bleeding and a baseline platelet count below 30.000 were identified as independent predictors for mortality (9). Currently, mortality is thought to be declining, which could be explained by the rising knowledge of TTS-associated CVST (8).

Patients vaccinated with ChAdOx1 nCov-19 (10) (AstraZeneca) and the Ad26.COV2.S vaccine (Johnson & Johnson/Janssen) (11) can develop TTS that is thought to cause CVST. TTS has not been described in mRNA vaccines. The criteria for TTS were lately defined as follows: (a) COVID vaccine (Johnson & Johnson/Astra Zeneca) 4 to 30 days previously, (b) venous or arterial thrombosis (often cerebral or abdominal), (c) thrombocytopenia, and (d) positive PF4 “HIT” ELISA (12). The antibodies against PF4-polyanion complexes are thought to cause massive platelet activation and thrombocytopenia. In this prothrombotic state, patients might develop CSVT. The mechanisms seem to be similar to HIT (10, 13, 14). In HIT, it is recommended to start the patients on intravenous immunoglobulins (1 g/kg body weight daily for 2 days) and anticoagulation with argatroban, bivalirudin, danaparoid, fondaparinux, or a direct oral anticoagulant at a therapeutic dose. Heparin should be avoided (14). There are currently no guidelines for the duration of anticoagulation. As to the diagnosis, in TTS-associated CVST, the necessary imaging is of course the same as in non-TTS-associated CVST.

Headaches are common after COVID-19-vaccination and are usually resolved the next day. The recurrence of unprecedented headaches raises the suspicion of CVST. However, even though both the patients complained of persisting headaches, CSVT was not detected by initial imaging performed on day 8 and day 12 after vaccination, respectively. At the time of marked thrombocytopenia, a follow-up MRI finally revealed thromboses. Therefore, performing a single investigation is not sufficient in patients with persistent headaches following ChAdOx1 nCov-19-vaccination. We hypothesize that the persisting headache following vaccination might represent a state of ongoing thrombosis and auto-thrombolysis in the cerebral sinus veins. In comparison, patients with “common” CVST complain of headaches when thrombosis is present (1).

In our surviving patient, partial recanalization occurred earlier as would be expected in non-TTS-associated CVST and was associated with normalization of the platelet count. The mechanism in TTS-associated CVST represents an immunological phenomenon opposed to non-TTS-associated CVST that is usually caused by a congenital or acquired coagulopathy. This might explain why treatment with immunoglobulins, which interrupts the immunological reaction, leads to a prompt onset of recanalization. This is also different from “common” CVST, in which patients usually have to be treated with anticoagulants for many months (1).

A similar case has been described by Ikenberg et al. (15) who reported a young woman complaining of headache 7 days after ChAdOx1 nCov-19-vaccination. The initial MRI did not show any changes. A second MRI, 3 days later, showed extensive CVST. TTS was confirmed and the patient recovered after treatment with intravenous immunoglobulins and argatroban (15).

The clinical course and treatment of the patients were similar to the already reported cases, apart from the inconclusive first imaging. Physicians must be aware of this phenomenon when treating patients with headaches following vaccination and should repeat imaging in patients with thrombocytopenia and elevated D-Dimer. People undergoing vector vaccination for SARS-Cov2 should be educated by the physician administering the vector vaccine to seek help in case of non-remitting headache.

The risk for “occult” vaccine-associated CSVT seems to be low and when detected, the current treatment regime is thought to be effective. Given the mortality of SARS-Cov2-infection with possible long-term effects, vaccination should continue unchanged (16).

## Conclusion

In patients with TTS complaining of persisting headache, a normal venous angiography might suggest a false sense of security for patients and treating physicians. Follow-up imaging several days later is necessary for patients with non-remitting headaches, to identify patients with CSVT and initiate the proper therapy to avoid a potentially fatal cause.

## Data Availability Statement

The original contributions presented in the study are included in the article/supplementary material, further inquiries can be directed to the corresponding author/s.

## Ethics Statement

Ethical review and approval was not required for the study on human participants in accordance with the local legislation and institutional requirements. The patients/participants provided their written informed consent to participate in this study.

## Author Contributions

TB, MJ, FR, HH, and MV treated the patients. TS performed and analyzed the CT and MRI results. FS performed the craniectomy. TB and FR wrote the manuscript. Each author approved the manuscript and contributed important intellectual content. All authors were involved in the analysis and interpretation of the findings, contributed to the writing, approved the final manuscript, read the ICMJE criteria for authorship, read, and agreed with the results of the study and conclusion.

## Conflict of Interest

The authors declare that the research was conducted in the absence of any commercial or financial relationships that could be construed as a potential conflict of interest.

## Publisher's Note

All claims expressed in this article are solely those of the authors and do not necessarily represent those of their affiliated organizations, or those of the publisher, the editors and the reviewers. Any product that may be evaluated in this article, or claim that may be made by its manufacturer, is not guaranteed or endorsed by the publisher.

## Abbreviations

CRP: C-reactive protein
CT: computed tomography
CVST: cerebral venous sinus thrombosis
GP: general practitioner
ICH: intracranial hemorrhage
HIT: heparin-induced thrombocytopenia
MRI: magnetic resonance imaging
PTT: partial thromboplastin time
SAH: subarachnoid hemorrhage
TTS: thrombotic thrombocytopenic syndrome.

## References

[1] Ferro JM Aguiar de Sousa D. Cerebral venous thrombosis: an update. Curr Neurol Neurosci Rep. (2019) 19:74. 10.1007/s11910-019-0988-x 31440838

[2] Ferro JM Bendszus M Jansen O Coutinho JM Dentali F Kobayashi A. Recanalization after cerebral venous thrombosis. A randomized controlled trial of the safety and efficacy of dabigatran etexilate versus dose-adjusted warfarin in patients with cerebral venous and dural sinus thrombosis. Int J Stroke. (2021). 10.1177/17474930211006303. [Epub ahead of print]. 33724104

[3] Kenet G Kirkham F Niederstadt T Heinecke A Saunders D Stoll M. Risk factors for recurrent venous thromboembolism in the European collaborative paediatric database on cerebral venous thrombosis: a multicentre cohort study. Lancet Neurol. (2007) 6:595–603. 10.1016/S1474-4422(07)70131-X 17560171 PMC1906729

[4] Krzywicka K Heldner MR van Sánchez Kammen M van Haaps T Hiltunen S Silvis SM. Post-SARS-CoV-2-vaccination cerebral venous sinus thrombosis: an analysis of cases notified to the European Medicines Agency. Eur J Neurol. (2021) 28:2656–3662. 10.1111/ene.15029 34293217 PMC8444640

[5] Schulz JB Berlit P Diener HC Gerloff C Greinacher A Klein C. COVID-19 vaccine-associated cerebral venous thrombosis in Germany. Ann Neurol. (2021) 90:627–39. 10.1101/2021.04.30.21256383 34288044 PMC8427115

[6] Sharifian-Dorche M Bahmanyar M Sharifian-Dorche A Mohammadi P Nomovi M Mowla A. Vaccine-induced immune thrombotic thrombocytopenia and cerebral venous sinus thrombosis post COVID-19 vaccination; a systematic review. J Neurol Sci. (2021) 428:117607. 10.1016/j.jns.2021.117607 34365148 PMC8330139

[7] van Sánchez Kammen M Heldner MR Brodard J Scutelnic A Silvis S Schroeder V. Frequency of thrombocytopenia and platelet factor 4/Heparin antibodies in patients with cerebral venous sinus thrombosis prior to the COVID-19 pandemic. JAMA. (2021) 326:332–8. 10.1001/jama.2021.9889 34213527 PMC8317004

[8] van de Munckhof A Krzywicka K Aguiar de Sousa D Sánchez van Kammen M Heldner MR Jood K. Declining mortality of cerebral venous sinus thrombosis with thrombocytopenia after SARS-CoV-2 vaccination. Eur J Neurol. (2021). 10.1111/ene.15113. [Epub ahead of print]. 34536256 PMC8652752

[9] Pavord S Scully M Hunt BJ Lester W Bagot C Craven B. Clinical features of vaccine-induced immune thrombocytopenia and thrombosis. N Engl J Med. (2021) 385:1680–9. 10.1056/NEJMoa2109908 34379914 PMC10662971

[10] Schultz NH Sørvoll IH Michelsen AE Munthe LA Lund-Johansen F Ahlen MT. Thrombosis and thrombocytopenia after ChAdOx1 nCoV-19 vaccination. N Engl J Med. (2021) 384:2124–30. 10.1056/NEJMoa2104882 33835768 PMC8112568

[11] Muir KL Kallam A Koepsell SA Gundabolu K. Thrombotic thrombocytopenia after Ad26.COV2.S vaccination. N Engl J Med. (2021) 384:1964–5. 10.1056/NEJMc2105869 33852795 PMC8063883

[12] James B Bussel MD Jean M Connors MD Douglas B Cines MD. Thrombosis with Thrombocytopenia Syndrome (also termed Vaccine-induced Thrombotic Thrombocytopenia). (2021). Available online at: https://www.hematology.org/covid-19/vaccine-induced-immune-thrombotic-thrombocytopenia (accessed June 29, 2021).

[13] Greinacher A Thiele T Warkentin TE Weisser K Kyrle PA Eichinger S. Thrombotic Thrombocytopenia after ChAdOx1 nCov-19 Vaccination. N Engl J Med. (2021) 384:2092–101. 10.1056/NEJMoa2104840 33835769 PMC8095372

[14] Furie KL Cushman M Elkind MS Lyden PD Saposnik G American Heart Association/American Stroke Association Stroke Council Leadership. Diagnosis and management of cerebral venous sinus thrombosis with vaccine-induced thrombotic thrombocytopenia. Stroke. (2021) 52:2478–82. 10.1161/STROKEAHA.121.035564 33914590

[15] Ikenberg B Demleitner AF Thiele T Wiestler B Götze K Mößmer G. Cerebral venous sinus thrombosis after ChAdOx1 nCov-19 vaccination with a misleading first cerebral MRI scan. Stroke Vasc Neurol. (2021). 10.1136/svn-2021-001095. [Epub ahead of print]. 34244448 PMC8717801

[16] Cao J Tu W-J Cheng W Yu L Liu Y-K Hu X. Clinical features and short-term outcomes of 102 patients with coronavirus disease 2019 in Wuhan, China. Clin Infect Dis. (2020) 71:748–55. 10.1093/cid/ciaa243 32239127 PMC7184479

